# Lateral column lengthening versus subtalar arthroereisis for pes planovalgus in patients with cerebral palsy: a systematic review and meta-analysis

**DOI:** 10.3389/fped.2024.1443447

**Published:** 2024-09-18

**Authors:** Chang-Hao Lin, Chun-Ho Chen, Shu-Hsin Yao

**Affiliations:** ^1^ Department of Orthopedics, Ditmanson Medical Foundation Chia-yi Christian Hospital, Chia-yi, Taiwan; ^2^ Department of Orthopedic Surgery, National Taiwan University Hospital, Taipei, Taiwan; ^3^ School of Medicine, National Taiwan University, Taipei, Taiwan

**Keywords:** cerebral palsy, flatfoot, pes planovalgus, lateral column lengthening, subtalar arthroereisis

## Abstract

**Introduction:**

Although pes planus, a common deformity in children with cerebral palsy (CP), is predominantly treated through lateral column lengthening (LCL), subtalar arthroereisis (SA) has also gained popularity for this purpose. This systematic review was conducted to compare surgical outcomes between LCL and SA for pes planovalgus in children with CP.

**Methods:**

PubMed, EMBASE, Cochrane Library, and Google Scholar were comprehensively searched for relevant articles reporting the outcomes of LCL and SA in the target population. Surgical outcomes were evaluated in terms of radiographic parameters and postoperative complications.

**Results:**

This review included 22 studies involving patients undergoing LCL (LCL group) and 9 studies involving those undergoing SA (SA group). LCL outperformed SA in terms of corrections in the talonavicular coverage angle (8.1°–42.1° vs. 8.0°–30.7°), anteroposterior talo–first metatarsal angle (12.3°–33.7° vs. 9.8°–21.4°), and calcaneal pitch angle (2.5°–29.7° vs. 3.5°–8.0°). Furthermore, the risk of postoperative complications, such as recurrence, pain, undercorrection, and overcorrection, was higher in the LCL group than in the SA group. However, the risks of reoperation and implant-related problems were higher in the SA group than in the LCL group. A meta-analysis of two randomized studies revealed that improvement in calcaneal pitch angle was significantly greater in the LCL group than in the SA group (mean difference: 2.09°; *P* = 0.0488).

**Conclusion:**

LCL outperforms SA in correcting pes planus–related radiographic parameters in patients with CP. However, postoperative complications appear to be more common after LCL than after SA.

**Systematic Review Registration:**

https://inplasy.com/inplasy-2024-5-0126, Identifier 202450126.

## 1 Introduction

Cerebral palsy (CP) is a neurological disorder that affects movement and posture, often leading to musculoskeletal deformities. Pes planovalgus, a common foot deformity in children with CP, is characterized by hindfoot valgus and longitudinal arch flattening, which can compromise gait and overall function (1). Surgical intervention is indicated when pain and dysfunction persist despite conservative treatment.

Several surgical methods are used to treat pes planovalgus (2). Although lateral column lengthening (LCL) is the predominant surgical option (2), subtalar arthroereisis (SA) has gained popularity because of its advantages, such as a low extent of invasiveness, reduced level of postoperative edema, early initiation of weight-bearing, short duration of hospitalization, and feasibility of associated soft tissue and bony procedures (3).

In a systematic review comparing clinical outcomes between LCL and SA for pediatric pes planovalgus, patients undergoing LCL achieved greater radiographic corrections and higher American Orthopaedic Foot and Ankle Society scores than did those undergoing SA. However, postoperative complications were more common after LCL than after SA (4). To the best of our knowledge, no systematic review has compared clinical outcomes between LCL and SA for pes planovalgus in children with CP. Although most studies on this topic have reported the outcomes of LCL and SA without any comparison, data synthesis can still provide valuable insights for surgeons. Therefore, this systematic review and meta-analysis was conducted to compare surgical outcomes—radiographic correction and postoperative complications—between LCL and SA for pes planovalgus in children with CP. For studies with a comparative design, a meta-analysis was also conducted.

## 2 Methods

### 2.1 Ethics and guidelines

This systematic review and meta-analysis was conducted following the Preferred Reporting Items for Systematic Reviews and Meta-Analyses guidelines. The protocol for this review was registered in the International Platform of Registered Systematic Review and Meta-analysis Protocols (registration number: 202450126).

### 2.2 Data sources and search strategy

Relevant articles were identified by systematically searching PubMed, EMBASE, Cochrane Library, and Google Scholar on January 17, 2024 by using free-text and Medical Subject Headings terms. The following terms were used for literature search: [(“flatfoot” OR “flatfeet” OR “pes planovalgus” OR “pes planus”) AND “cerebral palsy”] AND (“arthroereisis” OR “Evans osteotomy” OR “calcaneal lengthening osteotomy” OR “lateral column lengthening”).

### 2.3 Eligibility criteria and study selection

Two reviewers independently selected studies for inclusion in this systematic review. We included studies that reported the clinical outcomes of LCL or SA in children with CP and pes planovalgus. Protocols, case reports, reviews, comments, letters, and conference articles were excluded from the analysis. Publication date and language were not limited.

### 2.4 Data extraction and quality assessment

Two reviewers independently extracted the following data from the included studies: name of the first author; year of publication; level of evidence; type of surgery (LCL or SA); sample size, age, and sex of patients; duration of follow-up; outcomes of interest; flexibility of the feet; ambulatory status; Gross Motor Function Classification System (GMFCS) levels; and concomitant procedures. Surgical outcomes of interest were evaluated in terms of radiographic parameters and postoperative complications. The following radiographic parameters were assessed: talonavicular coverage, anteroposterior talo–first metatarsal, lateral talo–first metatarsal, anteroposterior talocalcaneal, lateral talocalcaneal, calcaneal pitch, and talo–horizontal angles. The following postoperative complications were assessed: recurrence, reoperation, postoperative pain, implant dislocation, implant fracture, implant-related problems, infection, temporary supination, undercorrection, overcorrection, neurovascular damage, fracture of the distal part of the calcaneus, calcaneocuboid joint subluxation, donor site morbidity, graft malposition, nonunion, and delayed union.

Two reviewers independently assessed the risk of bias in the included studies by using the methodological index for non-randomized studies tool (MINORS) for non-randomized studies and the Version 2 of the Cochrane risk-of-bias tool for randomized trials (RoB 2) (5, 6). Disagreements between the reviewers were resolved through discussion with a third reviewer until consensus was reached.

### 2.5 Statistical analysis

For continuous variables, we extracted mean and standard deviation values and calculated mean differences for between-group comparisons. For categorical variables, we extracted frequency and percentage values and calculated odds ratios for between-group comparisons. The included studies were divided into comparative studies and all studies. For comparative studies, a meta-analysis was performed using RevMan (version 5.4). Heterogeneity among the included studies was examined using the I^2^ statistic; a fixed-effects model was used when no significant heterogeneity was observed (*I*^2^ < 50%). Forest plots were generated to present the results of each study and the pooled effects of all included studies. The pooled effects were analyzed using the *z*-test. During the pooling of data from all included studies, the outcomes of interest were presented separately for LCL and SA.

## 3 Results

### 3.1 Search results

**Figure 1:**
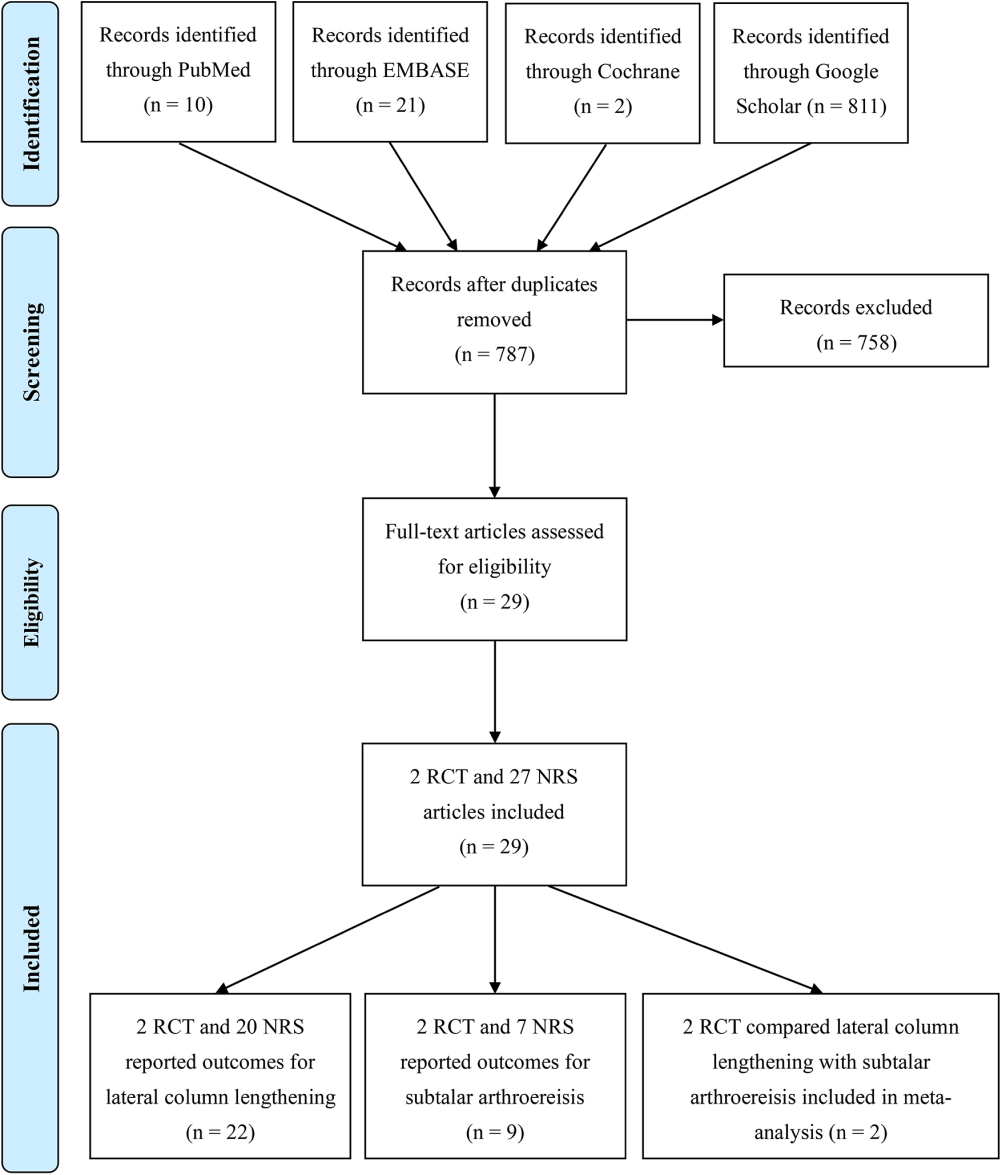
PRISMA flowchart depicting article selection.

Initially, the literature search returned 844 articles. After the removal of duplicates, 787 articles remained. After the application of the inclusion and exclusion criteria, 2 RCTs and 27 non-randomised studies (NRS) were assessed for eligibility. Among these 29 studies, 2 RCTs and 20 NRSs reported the outcomes of LCL, 2 RCTs and 7 NRSs reported the outcomes of SA, and 2 RCTs compared outcomes between LCL and SA (Figure 1).

### 3.2 Study characteristics and quality assessment

The characteristics of the included studies are presented in Table 1. In the 29 studies, patients' mean or median age ranged from 6 to 12.1 years. The minimum follow-up duration was <12 months in 3 studies, 12–24 months in 12 studies, and ≥24 months in 14 studies. Radiographic parameters were reported in 25 studies, recurrence was reported in 11 studies with a follow-up duration of ≥12 months, and postoperative complications were reported in 24 studies. Flexibility of the feet was reported in 13 studies, and all of the feet were flexible. Ambulatory status or GMFCS levels were reported in 25 studies, and most patients were ambulatory (Table 2). Concomitant procedures are presented in Table 3. The most common concomitant procedures were Achilles lengthening and gastrocnemius recession or lengthening. The next most common procedures were peroneus brevis and longus lengthening, which were performed in both groups. The scores of MINORS are presented in Table 4. The median scores were 12.5 for LCL-focused studies (range: 6–17) and 13 for SA-focused studies (range: 7–14), indicating that LCL-focused studies and SA-focused studies were similar in terms of quality. The RoB 2 based bias risk assessment table for RCTs are presented in Table 5.

**Table 1:** Summary of studies included in this review.

Study	Level of evidence	LCL or SA	Sample size (feet)	Mean or median age (years)	Men %	Follow-up period (months)	Outcomes for analysis
El Riheem et al. (7)	I	LCL vs. SA	LCL: 18 SA: 18	Range: 4–15		Mean: 11.5 Range: 6–18	1. Radiographic measurements 2. Complications
Ahmed et al. (8)	I	LCL vs. SA	LCL: 29 SA: 28	LCL: 9.1 SA: 9.0	LCL: 47.4% SA: 62.5%	Mean: 15.6 Range: 12–22	1. Radiographic measurements 2. Recurrence 3. Complications
Erdal et al. (9)	IV	LCL	86	11.6	60.0%	Mean: 42.6 Range: 22–92	1. Radiographic measurements 2. Recurrence 3. Complications
Rethlefsen et al. (10)	III	LCL	46	10.5	50.0%	Mean: 56.4 Minimum: 12	1. Complications
Narang et al. (11)	IV	LCL	17	11.13		Minimum: 12	1. Radiographic measurements 2. Recurrence 3. Complications
Aboelenein et al. (12)	IV	LCL	22	11.5	33.3%	Mean: 31 Range: 26–44	1. Complications
El-Hilaly et al. (13)	IV	LCL	18	9.7	55.6%	Mean: 4 Range: 2.3–6.1	1. Radiographic measurements
Aly et al. (14)	IV	LCL	24	10.74	56.3%	Mean: 33.5 Range: 24–48	1. Radiographic measurements 2. Complications
Cho et al. (15)	III	LCL	77	10.5	61.4%	Mean: 61.2 Range: 24–123.6	1. Radiographic measurements 2. Complications
Rhodes et al. (16)	III	LCL	63	9.3	55.6%	Range: 21.2–53.7	1. Recurrence 2. Complications
Luo et al. (17)	III	LCL	30	11.9	70.0%	Mean: 30 Range: 12–72	1. Radiographic measurements 2. Complications
Kadhim et al. (18)	III	LCL	15	11	46.7%	Mean: 130.8 Range: 75.6–184.8	1. Complications
Sung et al. (19)	IV	LCL	129	11	68.0%	Mean: 37.2 Range: 12–100.8	1. Radiographic measurements
Huang et al. (20)	III	LCL	37	11.02	38.1%	Mean: 29.4 Range: 12–63.7	1. Radiographic measurements 2. Complications
Kadhim et al. (21)	III	LCL	63	11.9	61.9%	Minimum: 12	1. Radiographic measurements 2. Recurrence 3. Complications
Adams et al. (22)	III	LCL	61	9.5	45.2%	Mean: 70 Range: 41–102	1. Radiographic measurements 2. Complications
Ettl et al. (23)	IV	LCL	28	8.6	63.2%	Mean: 51.6 Range: 12–103.2	1. Radiographic measurements 2. Recurrence 3. Complications
Park et al. (24)	III	LCL	37	8.1		Minimum: 26	1. Radiographic measurements
Zeifang et al. (25)	IV	LCL	46	11	68.8%	Mean: 66 Range: 36–108	1. Recurrence 2. Complications
Noritake et al. (26)	IV	LCL	27	10.8	62.5%	Mean: 38.4 Range: 24–60	1. Radiographic measurements 2. Recurrence 3. Complications
Yoo et al. (27)	IV	LCL	92	9.2		Mean: 62.4 Range: 24–93.6	1. Radiographic measurements 2. Recurrence 3. Complications
Andreacchio et al. (28)	III	LCL	23	10.2		Mean: 49.2 Range: 27.6–61.2	1. Radiographic measurements 2. Recurrence 3. Complications
Danilov et al. (29)	III	SA	18	Range: 7–16		Minimum: 24	1. Radiographic measurements
Elbarbary et al. (30)	IV	SA	46	8.6	69.6%	Mean: 36.7 Range: 24–40	1. Radiographic measurements 2. Recurrence
Kubo et al. (31)	III	SA	19	9.2	68.4%	Mean: 27.9 Range: 7–100	1. Radiographic measurements
Aleksandrov et al. (32)	III	SA	128	Range: 6–17		Range: 12–46	1. Radiographic measurements 2. Complications
Wen et al. (33)	III	SA	20	7.8	66.7%	Minimum: 20	1. Radiographic measurements 2. Complications
Silva and Fucs (34)	IV	SA	57	6	51.7%	Mean: 105 Range: 30–168	1. Radiographic measurements 2. Complications
Molayem et al. (35)	III	SA	27	12.1	46.7%	Mean: 50.4 Range: 26.4–75.6	1. Complications

LCL, lateral column lengthening; SA, subtalar arthroereisis.

**Table 2:** Flexibility and function of the feet in included studies.

Study	LCL or SA	Flexibility of the feet	Ambulatory status	GMFCS levels (% of patients or feet)				
				I	II	III	IV	V
El Riheem et al. (7)	LCL vs. SA	Flexible	Ambulatory	I, II: 100%				
Ahmed et al. (8)	LCL vs. SA	Flexible	Ambulatory	5.3%	68.4%	26.3%		
Erdal et al. (9)	LCL	Flexible		10.9%	50.9%	32.7%	5.5%	
Rethlefsen et al. (10)	LCL		Ambulatory	11.5%	46.2%	42.3%		
Narang et al. (11)	LCL		Ambulatory	70.6%	29.4%			
Aboelenein et al. (12)	LCL		Ambulatory		60%	40%		
El-Hilaly et al. (13)	LCL	Flexible	Ambulatory	5.6%	11.1%	27.8%	55.6%	
Aly et al. (14)	LCL	Flexible	Ambulatory	6.3%	18.8%	75%		
Cho et al. (15)	LCL			19.5%	36.4%	32.5%	11.7%	
Rhodes et al. (16)	LCL	Flexible		12.7%	20.6%	47.6%	12.7%	6.4%
Luo et al. (17)	LCL	Flexible			85%	10%	5%	
Kadhim et al. (18)	LCL		Ambulatory	I, II: 80%		III, IV: 20%		
Sung et al. (19)	LCL		Ambulatory					
Huang et al. (20)	LCL		Ambulatory		71.4%	23.8%	4.8%	
Kadhim et al. (21)	LCL		Ambulatory	I, II: 60.3%		III, IV: 39.7%		
Adams et al. (22)	LCL							
Ettl et al. (23)	LCL		Ambulatory 73.7% Nonambulatory 26.3%					
Park et al. (24)	LCL		Ambulatory					
Zeifang et al. (25)	LCL	Flexible	Ambulatory					
Noritake et al. (26)	LCL		Ambulatory					
Yoo et al. (27)	LCL	Flexible	Ambulatory					
Andreacchio et al. (28)	LCL	Flexible	Ambulatory					
Danlov et al. (29)	SA							
Elbarbary et al. (30)	SA	Flexible		21.7%	65.2%	13.0%		
Kubo et al. (31)	SA			10.5%	47.4%	31.6%	10.5%	
Aleksandrov et al. (32)	SA							
Wen et al. (33)	SA		Ambulatory					
Silva and Fucs (34)	SA	Flexible						
Molayem et al. (35)	SA	Flexible	Ambulatory					

LCL, lateral column lengthening; SA, subtalar arthroereisis; GMFCS, gross motor function classification system.

**Table 3:** Concomitant procedures in LCL and SA groups.

Concomitant procedures	LCL group	SA group
Achilles lengthening, gastrocnemius recession or lengthening	(7–10, 12, 14–28)	(7, 8, 29–33, 35)
Hamstring lengthening, hamstring tenotomy	(7–10, 13, 15, 23, 26, 28)	(7, 8)
Peroneus brevis or longus lengthening	(7, 10–14, 19, 24–28)	(7, 30, 31, 33)
Flexor hallucis longus lengthening	(17, 23, 26)	
Flexor digitorum longus lengthening		(26, 31)
Extensor digitorum longus lengthening	(17)	(31)
Tibialis anterior tendon transfer	(9)	(32)
Tibialis posterior tendon transfer	(9, 15)	
Peroneal transfer		(29)
Rectus transfer	(9, 23, 28)	
Hip muscle release	(10)	
Capsular imbrication of the talonavicular joint, talonavicular arthrolysis and reposition, Kidner procedure	(13, 23)	
Hallux valgus surgery	(9, 10, 15, 25)	
Adductor tenotomy	(7–9, 28)	

**Table 4:** Scores on the MINORS tool.

Study	LCL or SA	Study design	MINORS score	
			Total	Maximum
Erdal et al. (9)	LCL	Retrospective case-series study	7	16
Rethlefsen et al. (10)	LCL	Retrospective comparative study	13	24
Narang et al. (11)	LCL	Prospective case-series study	8	16
Aboelenein et al. (12)	LCL	Prospective case-series study	8	16
El-Hilaly et al. (13)	LCL	Prospective case-series study	12	16
Aly et al. (14)	LCL	Prospective case-series study	8	16
Cho et al. (15)	LCL	Retrospective case-control study	17	20
Rhodes et al. (16)	LCL	Retrospective comparative study	14	24
Luo et al. (17)	LCL	Retrospective case-control study	15	20
Kadhim et al. (18)	LCL	Retrospective comparative study	14	24
Sung et al. (19)	LCL	Retrospective case-series study	13	16
Huang et al. (20)	LCL	Retrospective comparative study	17	24
Kadhim et al. (21)	LCL	Retrospective comparative study	14	24
Adams et al. (22)	LCL	Retrospective comparative study	14	24
Ettl et al. (23)	LCL	Retrospective case-series study	7	16
Park et al. (24)	LCL	Retrospective comparative study	16	24
Zeifang et al. (25)	LCL	Prospective case-series study	10	16
Noritake et al. (26)	LCL	Case-series study	6	16
Yoo et al. (27)	LCL	Case-series study	6	16
Andreacchio et al. (28)	LCL	Case-series study	6	16
Danilov et al. (29)	SA	Comparative study	13	24
Elbarbary et al. (30)	SA	Prospective case-series study	8	16
Kubo et al. (31)	SA	Retrospective comparative study	13	24
Aleksandrov et al. (32)	SA	Comparative study	13	24
Wen et al. (33)	SA	Comparative study	13	24
Silva and Fucs (34)	SA	Retrospective case-series study	7	16
Molayem et al. (35)	SA	Retrospective comparative study	14	24

MINORS, methodological index for non-randomized studies; LCL, lateral column lengthening; SA, subtalar arthroereisis.

**Table 5:** Version 2 of the cochrane risk-of-bias tool for randomized trials.

Study	Experimental arm	Comparator arm	D1	D2	D3	D4	D5	Overall
El Riheem et al. 2023	LCL	SA	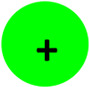	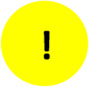	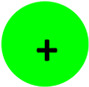	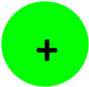	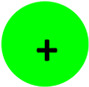	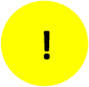
Ahmed et al. 2022	LCL	SA	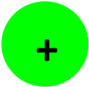	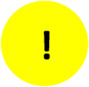	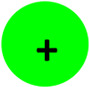	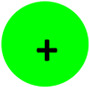	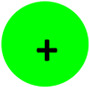	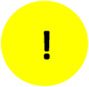
			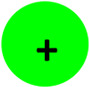	Low risk				
			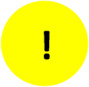	Some concerns				
			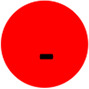	High risk				

LCL, lateral column lengthening; SA, subtalar arthroereisis; D1, randomization process; D2, deviation from the intended intervention; D3, missing outcome data; D4, measurement of the outcome; D5, selection of the reported result.

### 3.3 Clinical outcomes

#### 3.3.1 Radiographic parameters

**Figure 2:**
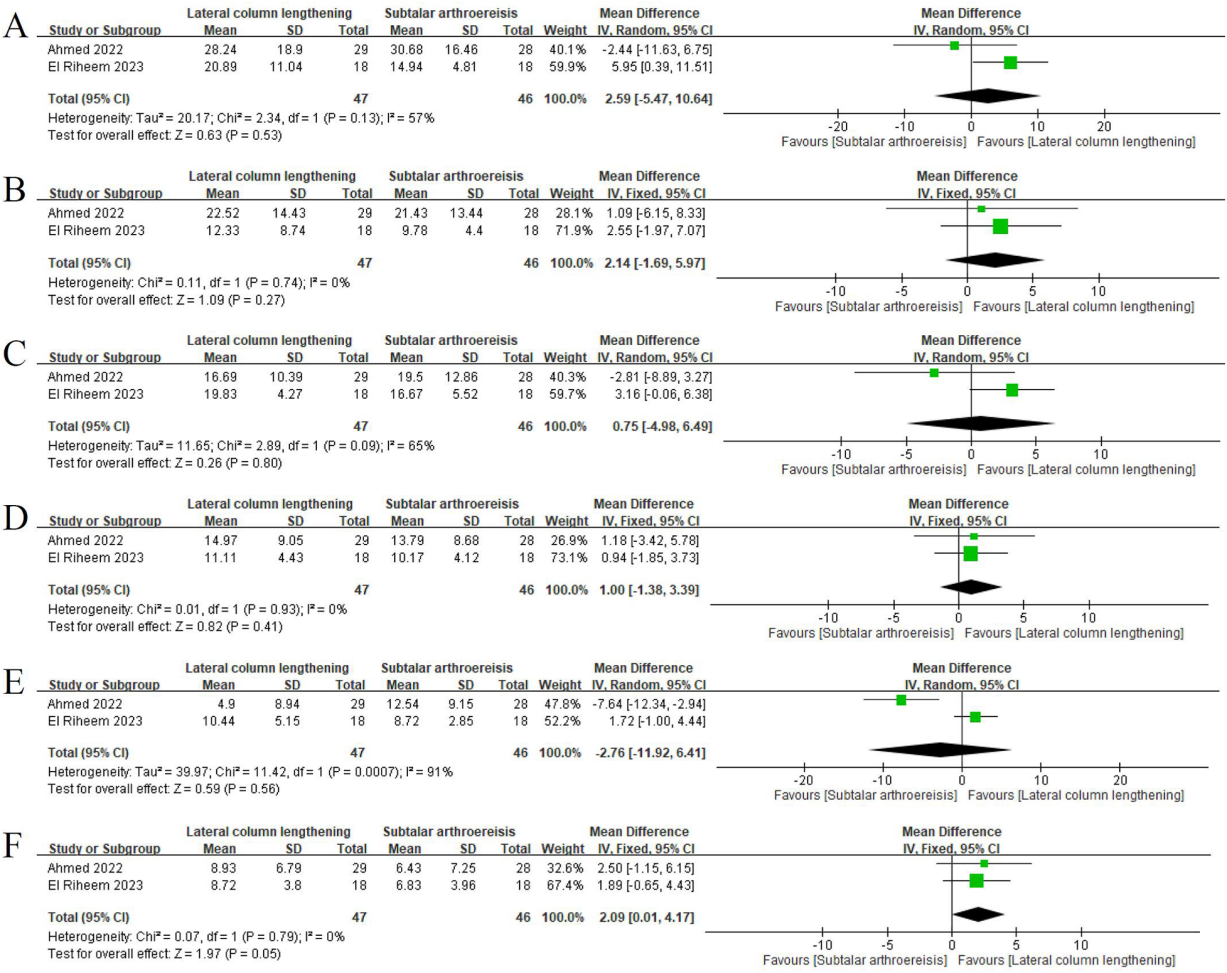
Forest plot for comparison between lateral column lengthening and subtalar arthroereisis. Improvements in the **(A)** talonavicular coverage, **(B)** anteroposterior talo–first metatarsal, **(C)** lateral talo–first metatarsal, **(D)** anteroposterior talocalcaneal, **(E)** lateral talocalcaneal, and **(F)** calcaneal pitch angles.

A total of 19 studies (905 feet) reported improvements in radiographic measurements for LCL and 8 (334 feet) for SA. Table 6 presents the range of mean values of seven radiographic parameters in patients undergoing LCL (LCL group) and those undergoing SA (SA group). The LCL group achieved greater corrections in the talonavicular coverage, anteroposterior talo–first metatarsal, and calcaneal pitch angles than did the SA group. In the meta-analysis of two RCTs (Figure 2) (7, 8), the pooled results revealed no significant between-group difference in improvement in the talonavicular coverage, anteroposterior talo–first metatarsal, lateral talo–first metatarsal, anteroposterior talocalcaneal, or lateral talocalcaneal angle. However, the improvement in the calcaneal pitch angle was significantly greater in the LCL group than in the SA group (mean difference: 2.09°; *P* = 0.0488).

**Table 6:** The range of mean values of radiographic measurements reported in the included studies.

	Lateral column lengthening				Subtalar arthroereisis			
Radiographic parameters	Preoperative	Postoperative	Final follow-up	Improvement (Post–Pre)	Preoperative	Postoperative	Final follow-up	Improvement (Post–Pre)
Talonavicular coverage angle	11.5–56.7	0–21.3	6.3–16.4	8.1–42.1	13.1–39.9	5.1–12.0	10.0^a^	8.0–30.7
Anteroposterior talo–first metatarsal angle	17.2–36.3	1.2–13.2	1.7–11.1	12.3–33.7	21.0–26.5	4.0–5.1	7.0^a^	9.8–21.4
Lateral talo–first metatarsal angle	3.0–33.9	2.7–18.4	4.8–20.2	0.3–19.8	21.6–38.0	1.1–15.0	15.0^a^	16.7–23.0
Anteroposterior talocalcaneal angle	22.7–34.7	19.2–29.0	15.4–27.6	5.6–15.0	27.0–46.2	18.3–36.3	17.0–36.2	2.9–16.4
Lateral talocalcaneal angle	21.7–49.8	17.1–41.5	23.4–44.5	−4.0–19.7	43.1–50.3	27.9–44.4	34.0–38.6	2.7–22.4
Calcaneal pitch angle	−1.1–13.8	5.3–28.6	10.2–17.3	2.5–29.7	3.1–7.7	9.5–14.1	10.1–13.0	3.5–8.0
Talo–horizontal angle	30.4–47.6	26.9–28.4	25.6–32.7	13.8–19.2	45.0^a^	28.0^a^	28.0^a^	17.0^a^

^a^ Reported by only one study.

#### 3.3.2 Postoperative complications

**Figure 3:**
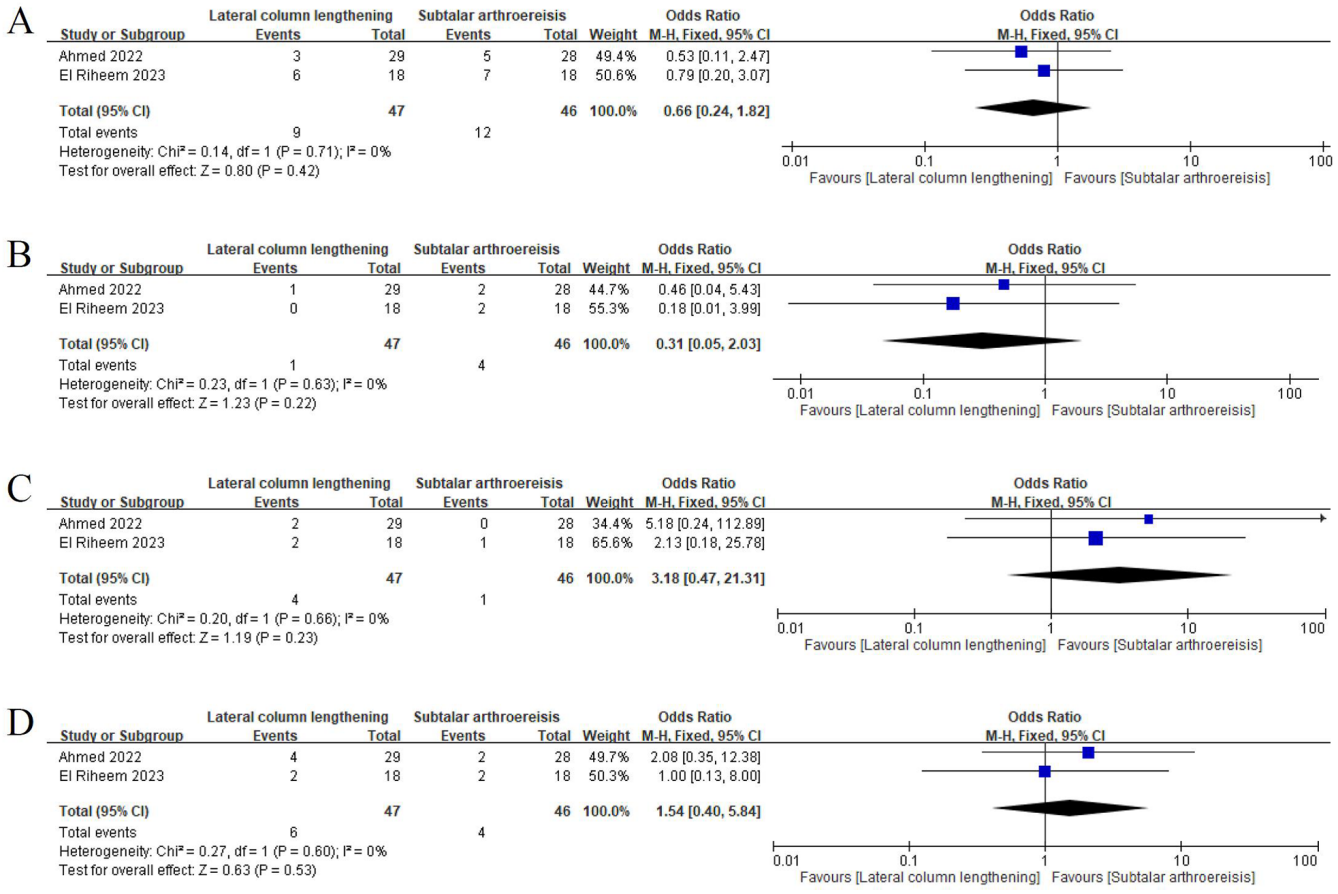
Forest plot for comparison between lateral column lengthening and subtalar arthroereisis. Incidence of pain **(A)** within the first 6 months after surgery and **(B)** 6 months after surgery. Incidence of **(C)** infection and **(D)** undercorrection.

The rate of each postoperative complication was pooled (Table 7) from 19 LCL-focused studies (804 feet) and 7 SA-focused studies (324 feet). The LCL group had considerably higher risks of recurrence, postoperative pain, undercorrection, and overcorrection than did the SA group. By contrast, the SA group had substantially higher risks of reoperation and implant-related problems than did the LCL group. In the LCL group, the rates of neurovascular damage, fracture of the distal part of the calcaneus, calcaneocuboid joint subluxation, calcaneocuboid joint arthrosis, donor site morbidity, graft malposition, nonunion, and delayed union were 0.6% (1/160), 0% (0/50), 6.3% (27/431), 1.2% (1/83), 0% (0/50), 0% (0/129), 0.9% (4/451), and 0.7% (2/276), respectively. A meta-analysis of two RCTs (7, 8) revealed no significant difference in the incidence of pain, infection, and undercorrection between the LCL and SA groups (Figure 3).

**Table 7:** Postoperative complications.

	Lateral column lengthening	Subtalar arthroereisis
Recurrence (≥12 months follow-up)	65/474 (13.7%)	0/74 (0.0%)
Recurrence (≥24 months follow-up)	20/188 (10.6%)	0/46 (0.0%)
Reoperation	11/212 (5.2%)	8/27 (29.6%)
Pain (≤6 months after surgery)	19/93 (20.4%)	14/194 (7.2%)
Pain (>6 months after surgery)	35/212 (16.5%)	6/194 (3.1%)
Implant-related problem^a^	6/63 (9.5%)	17/212 (8.0%)
Implant dislocation		15/212 (7.1%)
Implant fracture		2/27 (7.4%)
Infection	9/452 (2.0%)	2/112 (1.8%)
Temporary supination	0/47 (0.0%)	2/46 (4.3%)
Undercorrection	74/261 (28.4%)	4/46 (8.7%)
Overcorrection	20/291 (6.9%)	0/28 (0.0%)
Neurovascular damage	1/160 (0.6%)	0/20 (0.0%)
Fracture of the distal part of the calcaneus	0/50 (0%)	
Calcaneocuboid joint subluxation	27/431 (6.3%)	
Calcaneocuboid joint arthrosis	1/83 (1.2%)	
Donor site morbidity	0/96 (0.0%)	
Graft malposition	0/129 (0.0%)	
Nonunion	4/451 (0.9%)	
Delayed union	2/276 (0.7%)	

^a^ In the lateral column lengthening group, only studies that used staples, screws, and plates to fixate bone grafts were included.

## 4 Discussion

To the best of our knowledge, this review included the largest number of studies reporting the outcomes of LCL and SA for pes planovalgus in children with CP. Data from these studies were pooled and analyzed to provide comprehensive information on radiographic parameters and postoperative complication risks.

Most of the included studies focused on LCL. Over the years, SA has gained popularity because of its advantages, such as a low extent of invasiveness and early initiation of weight-bearing. A systematic review of studies on pediatric pes planovalgus reported that LCL outperformed SA in terms of radiographic corrections in the anteroposterior talo–first metatarsal angle (9.5°–21.7° vs. 10.6°–12.8°) and calcaneal pitch angle (2.1°–26.53° vs. −1.3°–3.23°) (4). Our review revealed similar results, with LCL outperforming SA in correcting the anteroposterior talo–first metatarsal, talonavicular coverage, and calcaneal pitch angles, likely because LCL involves adjusting the skeletal structure of the foot. However, our meta-analysis of two comparative studies indicated a significant between-procedure difference in the mean value of the calcaneal pitch angle. The inconsistency in our findings is likely attributable to the limited number of studies available for meta-analysis and the substantial heterogeneity observed in the talonavicular coverage, lateral talo–first metatarsal, and lateral talocalcaneal angles. While treating pes planovalgus in patients with cerebral palsy, performing single-event multilevel surgery according to the symptoms is common. Therefore, although concomitant procedures are associated with outcomes, conducting subgroup analysis is challenging because previous studies did not separately report outcomes for patients undergoing different combinations of procedures.

In the systematic review of studies on pediatric pes planovalgus, postoperative complications were more common after LCL than after SA (0%–86.9% vs. 3.5%–45%). However, the rate of reoperation was similar between the LCL and SA groups (0%–27.3% vs. 0%–36.4%) (4). We did not pooled data to calculate an overall rate of postoperative complications because the definitions of these complications varied across the included studies. The rate of postoperative complications may appear higher in studies presenting more comprehensive information on these complications. Therefore, we pooled data separately for each complication.

When comparing LCL and SA, we focused on complications reported for both LCL and SA. The risks of recurrence, postoperative pain, undercorrection, and overcorrection were substantially higher in the LCL group than in the SA group. By contrast, the risk of reoperation were higher in the SA group than in the LCL group. The elevated risk of recurrence in the LCL group may be associated with additional procedures for soft tissue, graft, and fixation. The increased incidence of postoperative pain after LCL may be attributable to its the relatively invasive nature of LCL. Undercorrection in LCL can result from inadequate intraoperative lateral column lengthening or subsequent loss of correction due to graft resorption. Premature weight-bearing, inappropriate graft composition, and insufficient fixation may also contribute to undercorrection. The increased risk of overcorrection in LCL may be associated with the sequence of medializing calcaneal osteotomy and LCL. Performing the medializing calcaneal osteotomy first can lead to overcorrection of the hindfoot deformity because of additional hindfoot inversion due to LCL (36). In the subtalar arthroereisis studies we included, detailed reports on implant-related problems were provided, but other complications, such as recurrence and undercorrection, were not necessarily mentioned. Therefore, potential bias may exist due to the insufficient information, which will require the inclusion of more SA studies to address this issue.

Patients with GMFCS levels III/IV had higher risks for undercorrection following LCL than those with GMFCS levels I/II in AP talus-first metatarsal angle and lateral talus-first metatarsal angle (15). A better satisfaction rate following LCL was also reported in patients with GMFCS levels II than those with GMFCS levels III/IV (20). Based on these results, for patients with GMFCS level III/IV, additional procedures should be considered when performing LCL. The subgroup analysis for GMFCS levels was not performed in this study because most of the included studies reported outcomes without stratifying by GMFCS levels and ambulatory status. Therefore, it is not possible to assess the association between GMFCS levels and outcomes in this study.

Among the 22 studies on LCL, only 6 reported the use of staples, screws, and plates for fixating bone grafts. However, implant-related problems were clearly reported in only one of these studies. The exact number of cases involving fixation was not reported clearly. Hence, we could not determine a reliable incidence of implant-related problems after LCL involving the use of implants. Further studies are needed to verify whether implants should be used in LCL to fixate bone grafts.

The rate of reoperation was higher in the SA group than in the LCL group. Among the included studies, only that of Molayem et al. reported the rate of reoperation after SA; the causes of reoperation were implant dislocation or fracture (35). The rate of implant-related problems was higher in the study of Molayem et al. (29.6%) than in those of Aleksandrov et al. (2.3%) and Silva et al. (10.5%) (32, 34, 35). Molayem et al. indicated that the high rate of implant-related problems was associated with performing SA without Achilles tendon lengthening to balance muscle forces around the joint (35).

Pooled results from our meta-analysis of two comparative studies revealed no significant difference between LCL and SA in the incidence of pain, infection, or undercorrection. The inconsistency in findings related to pain and undercorrection may be attributable to the limited number of studies available for meta-analysis. Moreover, not all included studies provided adequate information for distinguishing postoperative complications, thereby limiting the number of cases available for data synthesis. However, by exclusively including cases with clearly reported information on postoperative complications, we minimized the risk of overestimating or underestimating the rate of each complication.

Our study has several limitations. First, most of the included studies were case-series studies, because using a comparative design or performing randomization for the between-procedure comparison of clinical outcomes in the target population is a challenging task. Therefore, only limited data could be included in the meta-analysis. Moreover, although a comparative design was used in several studies, their objective was not to compare outcomes between LCL and SA, resulting in differences in outcomes of interest. This is particularly evident in SA studies, which tend to focus more on reporting implant-related outcomes than other parameters. Therefore, when comparing LCL and SA, there may be bias due to the insufficient information of included studies. Second, for some outcomes of interest, the sample size was small because of the lack of uniformity in the outcomes reported in the included studies. Third, although some studies have longer follow-up periods and maximum follow-up time points, they did not reported outcomes separately based on the length of the follow-up time. Therefore, we can only use the minimum follow-up time as the cutoff point to present the results. However, 2 years is a relatively short time for follow-up in CP patients. Finally, we could not perform subgroup analysis by potential confounding factors such as flexibility of the feet, GMFSC levels, ambulatory status, additional procedures, LCL site, graft types for LCL, and implant types for SA. The wide intra- and interstudy variations in these factors resulted in insufficient information for the separate evaluation of outcomes. In summary, our meta-analysis included studies of limited quality. Thus, caution should be exercised when interpreting our results. Although we included studies with relatively low levels of evidence, our pooled results provide valuable insights with clinical relevance.

In conclusion, in the treatment of pes planovalgus in children with CP, LCL may outperform SA group in terms of corrections in the talonavicular coverage, anteroposterior talo–first metatarsal, and calcaneal pitch angles. However, LCL is associated with increased risks of recurrence, postoperative pain, undercorrection, and overcorrection. By contrast, SA is associated with an elevated risk of reoperation, likely because of implant-related problems. These comparisons of complications are based on a limited number of SA studies.

## Data Availability

The raw data supporting the conclusions of this article will be made available by the authors, without undue reservation.
